# No evidence exists on outcomes of non-operative management in patients with femoroacetabular impingement and concomitant Tönnis Grade 2 or more hip osteoarthritis: a scoping review

**DOI:** 10.1007/s00167-022-07274-y

**Published:** 2022-12-09

**Authors:** Octavian Andronic, Leica Sarah Claydon-Mueller, Rachael Cubberley, Daniel Karczewski, Victor Lu, Vikas Khanduja

**Affiliations:** 1 Department of Orthopaedics, Balgrist University Hospital, University of Zürich, Forchstrasse, 340, 8008 Zurich, Switzerland; 2 Medical Technology Research Centre, Anglia Ruskin University, Bishop Hall Lane, Chelmsford, CM1 1SQ UK; 3 Young Adult Hip Service, Department of Trauma and Orthopaedics, Adden–brooke’s Cambridge University Hospital, Box 37, Hills Road, Cambridge, CB2 0QQ UK; 4 Department of Trauma and Orthopaedics, Center for Musculoskeletal Surgery, Charitè University Medicine Berlin, Charitéplatz 1, 10117 Berlin, Germany; 5 School of Clinical Medicine, University of Cambridge, Cambridge, CB2 0SP UK

**Keywords:** Femoroacetabular impingement, FAI, Hip osteoarthritis, Hip preservation, Non-operative, Outcomes

## Abstract

**Purpose:**

The purpose of this scoping review was to assess the outcomes of all the non-operative modalities of management for femoroacetabular impingement (FAI) and concomitant osteoarthritis (OA) Tönnis Grade 2 or more.

**Methods:**

A systematic search of PubMed was performed from inception to December 1st 2021 for literature on outcomes of non-operative management strategies for young adults with symptomatic FAI using the PRISMA Extension for Scoping Reviews guidelines. Cohorts investigating FAI and concomitant hip OA Tönnis Grade 2 or more were considered eligible. Studies not written in English or German, below level 4 evidence, and reviews were excluded. A secondary analysis for FAI without OA stratification was conducted after the initial screening to allow identification of available non-operative interventions.

**Results:**

No study reported outcomes separately for non-operative management of FAI with Tönnis Grade 2 OA or more and as such, did not fulfil the inclusion criteria.

A secondary analysis included 24 studies that reported on outcomes for non-operative interventions for FAI irrespective of the degree of degeneration. Three studies investigated the efficacy of hyaluronic acid injection, 5 reports investigated corticosteroid injections, 2 studies evaluated the outcomes of hip bracing and 16 studies included a physiotherapy programme. Associations between the aforementioned interventions were analysed.

There is level I evidence supporting the efficacy of activity modification and hip-specific physiotherapy for FAI and mild OA. Core-strengthening exercises are prevalent amongst successful regimens in the literature. Contradictory evidence questions the efficacy of hip bracing even for short-term outcomes. Corticosteroid injections have mostly failed in intention-to treat analyses but may be valuable in delaying the need for surgery; further studies are warranted. Reports on outcomes following hyaluronic acid injections are contradictory.

**Conclusion:**

No evidence exists on outcomes following non-operative management of FAI with concomitant Tönnis Grade 2 or more OA of the hip. Further studies are required and should explore the non-operative interventions that were employed for FAI and milder OA. There is strong evidence for a hip-specific physiotherapy program including activity modification and core strengthening exercises. Adjunct interventions such as corticosteroid injections and NSAID consumption may be valuable in delaying the need for surgery.

**Level of evidence:**

Level IV.

**Supplementary Information:**

The online version contains supplementary material available at 10.1007/s00167-022-07274-y.

## Introduction

Femoroacetabular impingement (FAI) represents an abnormal hip morphology that may be a cause of development of hip osteoarthritis (OA) in view of the abnormal mechanical stresses being placed on the joint [21, 37]. Usually, FAI is initially managed non-operatively [28], and this includes activity modification/reduction, physiotherapy and analgesia. If symptoms persist, joint-preservation surgery can be considered [2, 47]. However, outcomes of surgery for FAI with concomitant moderate- to advanced hip OA (Tönnis Grade ≥ 2) are equivocal, with some reports suggesting poor results and high rates of conversion to THA in the literature [13, 15, 22]. There is also concern that performing a joint-preservation procedure in this subgroup may lead to a rapid clinical decline that would require a THA sooner [52].

Previous randomized controlled trials (RCTs) (FASHIoN [24] and FAIT [45]) have successfully demonstrated good short-term clinical outcomes following non-operative management in patients with FAI and Tönnis Grade 0 and 1, even if it underperformed when compared to hip arthroscopy.

In a recent systematic review by our group [3, 4], inconclusive and contradictory results were found for outcomes of hip arthroscopy for FAI with Tönnis Grade 2 or more hip OA. Whilst a significant amount of hip preservation surgeons consider Tönnis Grade 2 to be a contraindication for hip preservation surgery [17], some experts [10, 11] reported favourable outcomes even in the context of moderate to advanced degeneration (Tönnis Grade 2 or more hip OA). These reports, however, did not include comparators with non-operative management.

In the context of a lack of consensus in the management of this specific patient cohort, the aim of the study was to investigate whether non-operative regimens alone may improve patient-reported outcomes (PROMs) and delay the need of subsequent surgery, either joint preservation or THA in patients with FAI and concomitant Tönnis Grade 2 or more hip OA.

## Materials and methods

### Identification of studies

The review followed the PRISMA Extension for Scoping Reviews (PRISMA-ScR) [50] guidelines and used the recommendations outlined by Arksey and O’Malley [8].

The literature search of the PubMed database included articles from inception to 1st of December 2021 using combinations of keywords as shown in Supplementary Table 1. Studies reporting on PROMs and clinical or radiographic outcomes of non-operative management for patients with FAI were included for analysis. Reviews, case reports, surgical techniques, oral presentations and letters were excluded from the analysis. Two reviewers independently screened all the studies (AO and DK). In the case of a discrepancy, the senior author (VK) was consulted.

For the primary analysis, studies investigating FAI and concomitant hip OA Tönnis Grade ≥ 2 were considered eligible. A secondary aim was to identify available non-operative interventions and their outcomes for FAI irrespective of the degree of degeneration including all types of classification and stages for OA.

The following exclusion criteria were employed: studies including participants with active inflammatory disease, neurologic conditions, previous ipsilateral surgeries of the hip, osteonecrosis or concomitant hip dysplasia.

### Data extraction

Data were extracted by two independent members of the review team. For each individual study of the secondary aim, information was recorded on an Excel Spreadsheet. The first author, name of the scientific journal, year of publication, and level of evidence according to the Oxford Centre for Evidence-Based Medicine 2011 Levels of Evidence [43] were extracted. Demographic and outcome data included the following: number of patients and hips, classification of osteoarthritis, Tönnis grading when available, indications, disease duration, type of intervention, age, body mass index (BMI), gender, length of follow-up, PROMs pre- and post-operative, radiological outcomes, rates of conversion to surgery or total hip arthroplasty, complications or adverse events and other interventions.

The extracted data were synthetized according to the level of evidence reported by the authors (Oxford Centre for Evidence-Based Medicine 2011 Levels of Evidence) [43] and type of non-operative intervention. At every step where there was a mismatch between reviewers during the screening and data extraction process, the senior author was contacted (VK) until agreement was reached.

## Results

### Study selection

**Fig. 1:**
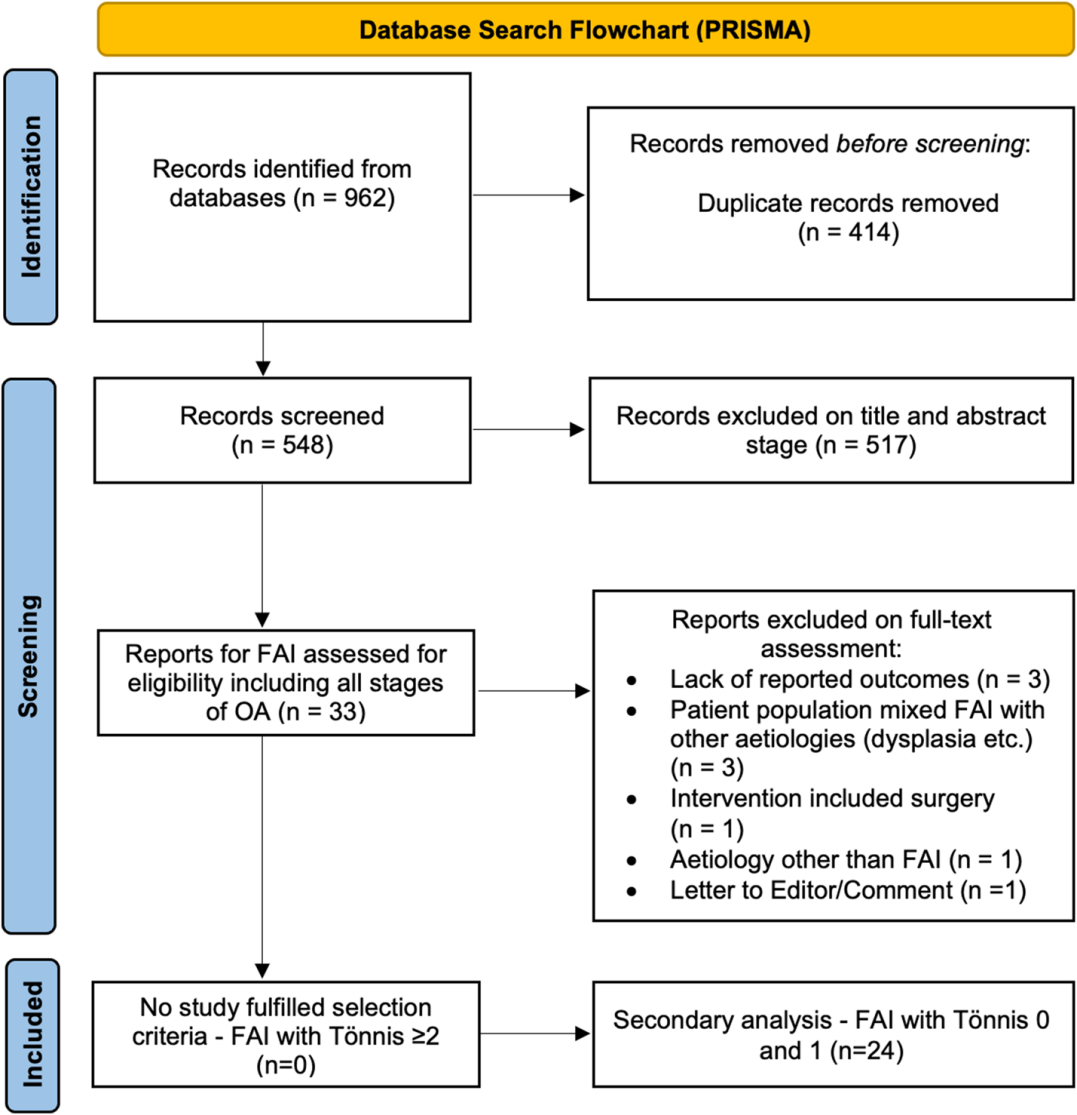
Flowchart of the scoping review

The database search yielded 962 studies. After removal of duplicates, a total of 548 articles were identified (Fig. 1). Five-hundred and seventeen studies were excluded at the title and abstract stage. Thirty-three full text papers were assessed for eligibility against the inclusion and exclusion criteria. Of these, not a single study met the inclusion and exclusion criteria. There was a single study that included patients with Tönnis Grade 2 OA, which however did not report outcomes for non-operative management separately for this cohort of patients [36].

The secondary analysis included all studies that reported outcomes for non-operative interventions for FAI irrespective of the degree of degeneration and type of classification for osteoarthritis. A total of 24 studies were included for the secondary analysis. Author-declared level of evidence was recorded. There were eight randomized controlled trials (RCTs) [20, 24, 27, 34–36, 45, 51], three prospective cohort studies [42, 46, 54], four retrospective case–control studies [7, 29, 30, 48] and nine retrospective case series [1, 12, 14, 19, 25, 32, 41, 44, 49].

### Hyaluronic acid injections

One RCT [34] and two retrospective cohort series [1, 44] utilised intra-articular hyaluronic acid (HAc) injections of the hip (Table 1) [1, 34, 44]. Abate et al. [1] also used activity restriction and allowed concomitant consumption of non-steroidal anti-inflammatory drugs (NSAIDs). The study reported slight improvement in PROMs (Table 1) at 12 months. Lee [34] compared hyaluronic acid injections (2 mL) with corticosteroid injections (20 mg triamcinolone acetate) and concluded after a mean follow-up of 3 months that no differences could be reported when separate injections were performed. In patients with crossover injection (both hyaluronic acid and corticosteroid), mean Hip disability Osteoarthritis Outcome Score (HOOS) increased from 51.7 at baseline to 71.0 at 3 months [34]. Ometti [44] registered significant improvement among all PROMs (visual analogue scale (VAS), Harris Hip Score (HHS), Lequesne) at 12 months after using a derivative of hyaluronic acid obtained by controlled chemical synthesis (2% partial hexadecylamide). Lee et al. [34] utilized fluoroscopic guidance as opposed to ultrasound guidance in the other two studies and were the only ones to report the dosages of injections.

**Table 1:** Hyaluronic acid injection

Study	LoE	Nr	OA staging	Indication	Age, years (range)	BMI (kg/m2)	Gender	Follow-up (months)	Disease duration (months)	Intervention	PROMs pre- to post-op	Conversion to surgery/THA	Other interventions
Abate 2014	IV	23	Absence of hip OA	FAI	45 ± 17 (26–7)	22.9 ± 1.6 (20–26.4)	13 M 7 F	12	7.8 ± 2.8 (3–12)	1 × hyaluronic acid injection + activity modification	mHHS: + 4.9; VAS: -5.0; Lequesne Index: -7.5	n.R	Concomitant consumption of NSAIDs
Lee 2016	I	16	Tönnis 0 and 1	FAI	37 (24–51)	n.R	11 M 19 F	3	n.R	Hyaluronic acid injection (2 mL)	In the group without crossover injection, mean HOOS showed no significant improvement and there was no significant difference according to injection drugs. In patients with crossover injection, mean HOOS increased from 51.65 at baseline to 71.02 at 12 weeks	n.R	Non-responders had one more hip injection at 2 weeks using alternative drug (crossover injection)
Control group		14	Tönnis 0 and 1	FAI		n.R		3	n.R	Corticosteroid injection (triamcinolone acetate 20 mg)		n.R	
Ometti 2020	IV	19	Tönnis 0 and 1	FAI	47 ± 5.3	23.3 ± 3.4	5 M 14 F	12	n.R	Intra-articular HYADD4-G injection	The variables VAS, HHS as well as Lequesne improved significantly at 12 months with the best improvement in the first 3 months. At the same time, a reduction in NSAIDs monthly intake was registered	n.R	Concomitant consumption of NSAIDs

*Nr* number of hips, *n.R*. not reported, *LoE* level of evidence, *OA* osteoarthritis, *BMI* body mass index, *FAI* femoroacetabular impingement, *M* male, *F* female, *PROMs* patient-reported outcomes, *THA* total hip arthroplasty, *HOOS* Hip disability Osteoarthritis Outcome Score, *mHHS* modified Harris Hip Score, *VAS* visual analogue scale, *NSAIDs* non-steroidal anti-inflammatory drugs, *HYADD4-G* hyaluronic hexadecylamide hyaluronic acid derivative

### Corticosteroid injections

One RCT [34], one prospective cohort study [46] and three retrospective case series [14, 32, 49] investigated the individual efficacy of corticosteroid injections either in a single cohort or compared to other interventions (Table 2). Lee et al. also investigated the outcomes of HAc injections and crossover injections, for both HAc and corticosteroid [34]. As mentioned previously, PROMs improved only for patients that underwent crossover injections at 3 months. Another comparative study [46] reported no significant differences in outcomes between the three interventions that were assessed (activity, physiotherapy and hip arthroscopy) at 2 years of follow-up (Table 2).

**Table 2:** Corticosteroid injection

Study	LoE	Nr	OA Staging	Indication	Avg. Age, years (range)	BMI (kg/m2)	Gender	Follow-up (months)	Disease duration (months)	Intervention	PROMs pre- to post-op	Conversion to Surgery/THA	Other interventions
Cianci 2019	IV	46	n.R	Labral tear with FAI	n.R. (14–20)	n.R	11 M 35 F	n.R	n.R	52 (68%) physiotherapy 55 (72%) 1 × corticosteroid injection 43 (57%) both treatments	39 (51%) resulted in surgical intervention	n. R	n.R
Krych 2014	IV	54	Tönnis 0 and 1	FAI	32 ± 12	n/a	19 M 35 F	n.R	n.R	Corticosteroid injection	Absolute change in NRS scores: 0 (0–8). At 6 weeks, only 3 patients reported significant pain relief (6%). 30 (88%) reported zero pain relief at 14 days post-injection	100% hip arthroscopy with a mean interval from injection to surgery of 119 (range 21–581) days	n.R
Lee 2016	I	16	Tönnis 0 and 1	FAI	37 (24–51)	n.R	11 M 19 F	3	n.R	Hyaluronic acid injection (2 mL)	In the group without crossover injection, mean HOOS showed no significant improvement and there was no significant difference according to injection drugs. In patients with crossover injection, mean HOOS increased from 51.65 at baseline to 71.02 at 12 weeks	n.R	Non-responders had one more hip injection at 2 weeks using alternative drug (crossover injection)
Control group		14	Tönnis 0 and 1	FAI		n.R		3	n.R	Corticosteroid injection (triamcinolone acetate 20 mg)		n.R	
Pennock 2018	II	65	n/a	FAI and labral tears	15.1 ± 2.0 (10.4–21.4)	n.R	23 M 42F	25.7 ± 6.7 (12.2–45.5)	10.8 6 17.2 (0.25–84)	Activity modification	No significant differences were noted in the proportion of patients meeting the MCID in the mHHS among the 3 treatment groups	n.R	n.R
Controls (1)		11	n/a	FAI and labral tears	16.6 ± 2.0 (13.6–21.4)	n.R	4 M 7F	25.4 ± 8.8 (11.7–40)	17.9 6 17.9 (1–60)	Corticosteroid injection		n.R	n.R
Controls (2)		17	n/a	FAI and labral tears	15.4 ± 0.9 (13.4–17.2)	n.R	6 M 11F	31.8 ± 12 (12.3–49.7)	5.1 6 4.5 (0.5–12)	Hip arthroscopy		n.R	n.R
Tangtiphaiboontana 2018	IV	9	n.R	FAI and labral tears	15.4 ± 1.1	n.R	1 M 8 F	29.4 (range 12–52)	n.R	Corticosteroid injection	52% (10/19 hips) went on to surgery after the injection	Hip arthroscopy (52%) at 12.8 months (range 2–36)	Physical therapy

*Nr* number of hips, *Avg* average, *n.R.* not reported, *n/a* not available or extraction not possible, *LoE* level of evidence, *OA* osteoarthritis, *BMI* body mass index, *FAI* femoroacetabular impingement, *M* male, *F* female, *PROMs* patient-reported outcomes, *THA* total hip arthroplasty, HOOS Hip disability Osteoarthritis Outcome Score, *mHHS* modified Harris Hip Score, *VAS* visual analogue scale, *MCID* minimal clinical important difference, *NRS* numeric rating scale

Krych et al.[32] reported a 100% rate of conversion to hip arthroscopy in all 54 hips treated with corticosteroid injections at a mean time of 3 months post-intervention. High rates of conversion have also been reported in the two remaining studies [14, 49], averaging 50% (Table 2).

### Hip bracing

Two studies with prospectively collected data (1 randomized controlled trial (RCT) [20] and 1 prospective cohort study) [42] evaluated the efficacy of a hip brace during a short period of follow-up (6 and 4 weeks, respectively). These have reported contradictory changes in PROMs (Table 3). Whilst Newcomb et al. [42] did not observe any significant changes in PROMs when compared to the standard care without the wearing of a hip brace, Eyles et al. [20] reported improvements for the HAGOS (The Copenhagen Hip and Groin Outcome Score) (p = 0.02) in the following categories: pain, symptoms, function and quality of life (QoL). However, these patients also received concomitant non-operative interventions with corticosteroid injections and oral NSAID consumption (Table 3).

**Table 3:** Hip bracing

Study	LoE	Nr	OA staging	Indication	Avg. age, years (range)	BMI (kg/m2)	Gender	Follow-up (months)	Disease duration (months)	Intervention	PROMs pre- to post-op	Conversion to surgery/THA	Other interventions
Newcomb 2017	II	17	n.R	FAI	27.1 ± 5.3	24.4 (2.6%)	17 M 8 F	1	3.9 ± 3.4	Hip brace (4 h/d for 4 weeks) + physiotherapy	Bracing over 4 weeks did not significantly change patient-reported outcomes (NRS pain, iHot-33 and HAGOS questionnaires)	n. R	n.R
*Control group*		8	n.R	FAI						Physiotherapy			
Eyles 2021	I	19	Tönnis 0 and 1	FAI and labral tears	38	25.5	8 M 11 F	1.5	n.R	Usual care + hip brace (6 weeks, 2 h/d week 1, up to 4 h/d week 2, and more than 4 h/d week 3 to 6) + physiotherapy (*n* = 6), corticosteroid (*n* = 1) injection; NSAIDs (*n* = 3)	Hip QoL improved in the brace group (*p* = 0.03); HAGOS (pain, symptoms, function and QoL) improved in the brace group (*p* = 0.02)	n.R	Similar rates of previous NSAIDs and corticosteroid injections between groups
*Control Group*		19	Tönnis 0 and 1	FAI and labral tears	41	25	7 M 12 F	1.5	n.R	Usual Care + Physiotherapy (*N* = 4), NSAIDs (*N* = 4)		n.R	

*Nr* number of hips, *Avg* average, *n.R*. not reported, *h/d* hours per day, *LoE* level of evidence, *OA* osteoarthritis, *BMI* body mass index, *FAI* femoroacetabular impingement, *M* male, *F* female, *PROMs* patient-reported outcomes, *THA* total hip arthroplasty, *HAGOS* The Copenhagen Hip and Groin Outcome Score, *QoL* quality of life, *NSAIDs* non-steroidal anti-inflammatory drugs

### Physiotherapy

Most studies that reported outcomes for non-operative interventions for FAI utilised different regimes of physiotherapy with or without other treatments (Table 4). Out of the 16 studies in this subgroup [7, 12, 19, 24, 25, 27, 29, 30, 35, 36, 41, 45, 46, 48, 51, 54], 5 randomized prospective studies [24, 27, 35, 36, 45] provided evidence for significant improvements post-intervention after hip-specific physiotherapy for patients with FAI, although underperforming when compared to hip arthroscopy (Table 4). There is also consistent reporting of significant positive impact on outcomes when employing core strengthening and postural exercises. A detailed breakdown of the exercise regimens and interventions is provided in Suppl. Table 2. The majority of studies also utilised activity modification with avoidance of positions with increased hip stress or restriction with appropriate patient education (Table 4). Seven studies [7, 12, 24, 25, 27, 29, 54] allowed concomitant interventions to be employed as a part of the non-operative regimen (corticosteroid injections and oral consumption of NSAIDs). Narveson et al. [41] emphasized the importance of therapeutic neuroscience education for patients with biopsychosocial issues.

**Table 4:** Physiotherapy

Study	LoE	Nr	OA staging	Indication	Avg. age, years (range)	BMI (kg/m2)	Gender	Follow-up (months)	Disease duration (months)	Intervention	PROMs pre to post-op	Conversion to surgery/THA	Other interventions
Casartelli 2019	IV	28	Tönnis 0	FAI	25 ± 5	23 ± 4	7 M 13 F	4	40 ± 36	Activity modification Hip-specific strengthening, core stability, postural balance exercises	GTO: 9 (29%) much better; 7 (22%) better; 6 (19%) somewhat better; 5 (16%) unchanged; 4 (14%) worse. Total 16 (52%) better; 11 (55%) responders, 9 (45%) non-responders	8 (25%) had hip surgery	Concomitant consumption of NSAIDs
Emara 2011	IV	37	Absence of hip OA	FAI	33 (23–47)	n.R	27 M 10 F	24	18.5 (9–36)	Physiotherapy Adaptation to pain-free ROM modification of daily activities	mHHS improved (*p* < 0.01); NAHS improved (*p* < 0.01); VAS improved (*p* < 0.01)	4 (11%) resulted in surgical intervention	n.R
Aoyama 2019	III	10	Tönnis 0 and 1	FAI	43.3 (31–54)	n.R	10 F	2	6 (1 – 18)	Pelvic floor muscle; trunk training; activity modification	Significantly better improvement in study group with trunk exercises for Vail hip score and iHOT12 (p < 0.001)	n.R	Concomitant consumption of NSAIDs
Control group		10	Tönnis 0 and 1	FAI	45.8 (29–54)	n.R	10 F	2	15 (1 – 25)	Pelvic floor muscle; activity modification		n.R	
Pennock 2018	II	65	n/a	FAI and labral tears	15.1 ± 2.0 (10.4–21.4)	n.R	23 M 42F	25.7 ± 6.7 (12.2–45.5)	10.8 6 17.2 (0.25–84)	Activity modification	No significant differences were noted in the proportion of patients meeting the MCID in the mHHS among the 3 treatment groups	n.R	n.R
Controls (1)		11	n/a	FAI and labral tears	16.6 ± 2.0 (13.6–21.4)	n.R	4 M 7F	25.4 ± 8.8 (11.7–40)	17.9 6 17.9 (1–60)	Corticosteroid injection		n.R	n.R
Controls (2)		17	n/a	FAI and labral tears	15.4 ± 0.9 (13.4–17.2)	n.R	6 M 11F	31.8 ± 12 (12.3–49.7)	5.1 6 4.5 (0.5–12)	Hip arthroscopy		n.R	n.R
Guenther 2017	IV	20	Absence of hip OA	FAI	29.8 ± 6.8	24.1 ± 2.9	2F, 18 M	3	53.6 ± 40.2 (12—132)	Physiotherapy. core exercises	HOOS: pain: + 8.5 (*p* = 0.003); symptoms: + 7.9 (*p* = 0.022); ADLs: + 10.4 (*p* = 0.003); sports: + 11.7 (*p* = 0.003); QOL: + 7.6 (*p* = 0.025)	Foreplaned surgery in all patients, 5/19 (26%) cancelled upcoming surgery after programme	Oral medication, massage treatments
Griffin 2018	I	195	Tönnis 0 and 1	FAI	35.2 ± 9.4	n.R	113 M 64 F	12	40	Individualized physiotherapy; activity modification	iHOT33 improvement greater in HA group (*p* = 0.009), no difference in other PROMs. More adverse events related to intervention in HA group (*p* = 0.017). Physiotherapy more cost-effective at 12 months in the setting of UK costs	14 (8%) received hip arthroscopy	Corticosteroid Injection
Control group		182	Tönnis 0 and 1	FAI	35.4 ± 9.7	n.R	100 M 71 F	12	37	Hip arthroscopy		1 (1%) converted to THA after hip joint infection	None
Hunter 2021	I	50	Tönnis 0 and 1	FAI	32,9	n.R	26 M 24 F	12	18 (2.5–120)	Physiotherapy	Hip-related quality of life (iHOT-33) showed a statistically and clinically important improvement in arthroscopy of 14 units (*p* = 0.003)	n.R	Injections
		49	Tönnis 0 and 1	FAI	32,9	n.R	31 M 18 F	12	24 (2–84)	Hip arthroscopy		n.R	n.R
Kekatpure 2017	III	54	n.R	FAI	47.9 ± 12	n.R	38 M 16 F	27.5	6.4 ± 8	Physiotherapy	The nonsurgical group had significant improvements in all clinical scores at the end of follow-up (p < 0.001)	44 hips underwent hip arthroscopy (45.4%) at 10 months (3 – 29.5)	Activity modification NSAIDs
*Control group*		44	n.R	FAI	41.8 ± 12	n.R	28 M 16 F	25.4	6.1 ± 5	Hip arthroscopy			
Kemp 2016	III	17	n.R	FAI	37 ± 8	25.1 ± 3.7	5 M 12 F	n.R	n.R	Personalized FAI-specific physiotherapy	All scores experienced improvement with more magnitude in the specialized group (iHOT-33: 27 ± 26; HOOS-QoL: 22 ± 18; HOOS pain: 20 ± 16)	n.R	n.R
Control group		7	n.R	FAI	38 ± 10	26.1 ± 2.4	12 M 5 F	n.R	n.R	Standard stretching therapy		n.R	n.R
Mansell 2018	I	40	≥ 2 cm joint space width on radiographs	FAI and labral tears	30.6 ± 7.4 (20–50)	27.47 ± 4.29	26 M 14 F	24	21 (53%) had over 2 years symptoms	Physiotherapy	Statistically significant improvements were seen in both groups on the HOS and iHOT-33, but the mean difference was not significant between the groups at 2 years	28 (70%) underwent surgery at a mean of 6.5 months	n.R
Control group		40	≥ 2 cm joint space width on radiographs	FAI and labral tears	29.7 ± 7.4 (21–44)	28.23 ± 4.39	21 M 19 F	24	22 (55%) had over 2 years symptoms	Hip Arthroscopy		Hip fracture (*n* = 1); heterotopic ossification (*n* = 1); revision surgery (*n* = 5); THA (*n* = 1)	n.R
Martin 2021	I	44	Tönnis 0, 1 and 2	FAI and labral tears	49.1 (47.7–50.6)	26.8 (25.6–28.0)	20 M 24 F	12	n.R	Physiotherapy	Intention-to-treat analysis revealed significantly greater iHOT-33 scores (112.11; *P* = 0.007) and mHHS scores (16.99 points; *P* = 0.04) in the surgical group than the physiotherapy group at 12 months	28 (63.6%) patients went on with hip arthroscopy at a mean of 6 months (4 – 8)	n.R
*Control group*		44	Tönnis 0, 1 and 2	FAI and labral tears	49.6 (47.7–51.5)	27.1 (25.8–28.4)	23 M 23 F	12	n.R	Hip arthroscopy		n.R	Postoperative physical therapy
Narveson 2018	IV	6	n.R	FAI and labral tears	37.8 ± 15.4 (20–65)	23.2 ± 6.86	1 M 5 F	2.5 (2 – 3)	26 ± 23.6	Physiotherapy	Clinically important improvements were seen on all self-reported outcome measures (iHOT – 33, NRS and other)	0 at 24 months	Therapeutic neuroscience education (for biopsychosocial issues)
Palmer 2019	I	110	Kellgren–Lawrence 0 and 1	FAI	36 ± 9.9	26.6. ± 4.8	37 M 73 F	8	n.R	Physiotherapy activity modification	After adjusting for baseline HOS ADL, age, sex, and study site, the mean HOS ADL was 10.0 points higher (6.4 to 13.6) in the arthroscopic hip surgery group compared with the physiotherapy programme group (*p* < 0.001))	2 (2%) at 8 months	None
Control group		112	Kellgren–Lawrence 0 and 1	FAI	36.4 ± 9.6	25.9 ± 4.8	38 M 74 F	8	n.R	Hip arthroscopy		None	Postoperative physical therapy
Spencer 2017	III	36	n.R	FAI and labral tears	40.0 (18–58)	27.9 (20.0–40.4)	15 M 21 F	20 (12–30)	n.R	Physiotherapy, corticosteroid injection or both	Mean mHHS scores similarly showed little change in the non-operative group (P¼ 0.91), and improved in the HA group (*p* < 0.001). At final follow up, mean NAHS scores after HA were significantly higher than scores for waitlist patients (*p* < 0.001)	n.R	n.R
Control group		36	n.R	FAI and labral tears	40.0 (18–58)	27.1 (20.3–37.0)	15 M 21 F	18 (12–36)	n.R	Hip arthroscopy		n.R	n.R
Wright 2016	I	7	n.R	FAI and labral tears	31 ± 4.9	25.6 ± 3.7	3 M 1F	2	n.R	Manual therapy and supervised exercise. Advice and home exercise	The between group differences for changes in pain or physical were not significant. Both groups showed statistically significant improvements in pain	Eight out of 15 (53%) patients elected to proceed with surgery	n.R
*Control group*		8	n.R	FAI and labral tears	36.1 ± 11.8	24.1 ± 7.4	1 M 7 F	2	n.R	Advice and home exercise alone			n.R
Zogby 2021	II	50	n.R	FAI and labral tears	15 ± 1.6	n.R	15 M 30 F	61.5 ± 8.2 (43.4–74.9)	11.7 ± 17.8 (0.25–84)	Physiotherapy, activity modification	Hips treated with activity modification and physical therapy alone met the MCID for the mHHS at a rate of 74% compared with a 71% rate for hips treated with an injection, and a 75% rate for hips treated with arthroscopic surgery. No difference in the proportion of hips that met the MCID for the mHHS based on treatment course was observed (*p* = 0.99)	12 (24%) underwent hip arthroscopy at a mean of 9.2 months (range, 1.3–18.1)	Corticosteroid injection (*n* = 7)
Control group		12	n.R	FAI and labral tears	15.4 ± 0.9	n.R	3 M 9 F	62.3 ± 7 (45.5–76.4)	5.7 ± 4.9 (0.5–12)	Hip arthroscopy		n.R	Activity modification involved discontinuation of all sports and activities that involved running, jumping, or high hip flexion

*Nr* number of hips, *Avg* average, *n.R*. not reported, *n/a* not available or extracted not possible, *LoE* level of evidence, *OA* osteoarthritis, *BMI* body mass index, *FAI* femoroacetabular impingement, *M* male, *F* female, *PROMs* patient-reported outcomes, *THA* total hip arthroplasty, *GTO* global treatment outcome, *HOOS* Hip disability Osteoarthritis Outcome Score, *mHHS* modified Harris Hip Score, *VAS* visual analogue scale, *iHOT* International Hip Outcome Tool, *NRS* numeric pain rating scale, *ROM* range of motion, *NSAIDs* non-steroidal anti-inflammatory drugs, *MCID* minimal clinically important difference, *QoL* quality of life, *ADL* activity of daily living.

There was a significant variability among reports regarding the proportions of patients who ultimately underwent surgery, ranging from 8 [24] to 70% [35] (Table 4). The average length of follow-up was heterogeneous and ranged from 2 [7] to 60 months [54]. Time to surgery was an equally heterogeneous parameter, with three studies reporting a mean time to surgery within 1 year after starting non-operative management (Kekatpure[29]–10 months (range, 3–29.5); Martin[36]–6 months (range, 4–8); Zogby[54] – 9.2 months (range, 1.3–18.1)).

## Discussion

The main purpose of this scoping review was to identify studies that reported on the outcomes of non-operative management options for FAI with concomitant Tönnis Grade 2 or more hip OA. A significant number of studies were excluded whereby the primary aetiology was not described or in cases of concomitant hip dysplasia. Only Martin et al. [36] evaluated outcomes of non-operative management which included hips with FAI and Tönnis 2 OA. The authors, however, did not report outcomes separately for this patient cohort, but instead cumulatively with Tönnis 0 and 1 OA, precluding data extraction and analysis. As such, we conclude that there is currently no evidence on outcomes of non-operative interventions for FAI with Tönnis Grade 2 or more hip OA. Along with the findings of a recent systematic review that investigated the outcomes of hip arthroscopy [3] for the same patient cohort, our findings demonstrate the lack of reliable evidence that would guide clinicians in treating these patients. Additionally, there may have been studies that have reported the outcomes of non-operative management of such patients, but lacked disease stratification and may have used hip osteoarthritis as their indication [26]. The majority of studies describing non-operative management for hip OA do not describe the primary aetiology [9, 23] that did not allow the inclusion of such studies. These studies may have provided valuable data, but due to increased selection bias and questionable primary aetiology (FAI/dysplasia or other causes of hip OA), we were not able to include them in the scoping review.

The secondary aim of the scoping review was to explore all possible non-operative interventions for the population of interest. There is level I evidence with large-scale RCTs that have shown achievement of good PROMs after hip-specific physiotherapy and activity modification for FAI and mild OA [24]. Nevertheless, these studies had a relatively short follow-up time for up to 12 months. A recent study by Zogby et al. [54] has demonstrated that non-operative management can achieve mid- to long-term symptom improvement and significantly delay the need for subsequent surgery for patients with FAI and labral tears. There is also evidence pointing to the importance of implementing core strengthening and postural exercises in the physiotherapy regimen [25]. Casartelli et al. [12] and Aoyama et al. [7] compared two cohorts, one with standard physiotherapy focusing on hip stretching and hip muscle strengthening and the other that included core strengthening and found significant improvements in PROMs in the latter. Physiotherapy may also have a beneficial effect if patients have concomitant causes of extraarticular hip pain such as iliopsoas impingement [5] or iliotibial band tightness or snapping [6], by means of muscle stretching and improvement in muscle balance. The prevalence of concomitant extraarticular conditions (ischiofemoral [39], iliopsoas and subspine impingement [40]) in FAI and the role of physiotherapy in this cohort needs to be further investigated. An important adjunct to a successful physiotherapy programme is appropriate patient education. Apart from systematic in-person supervised training sessions, the authors [30, 51] have emphasized the importance of patient education for home exercises and also techniques to increase patient compliance by reviewing patient progress using phone interviews or a training diary. Furthermore, Narveson et al. [41] have underlined the importance of additional therapeutic neuroscience education in patients that had biopsychosocial issues. A similar approach for hip arthroscopy, whereby surgeons were encouraged to screen patients for mental health disorders before surgery and offer them appropriate counselling, was described in a recent systematic review [16] due to associated poor surgical outcomes [33]. A therapeutic approach in this direction is currently lacking with only prognostic relationships being described.

Inconclusive and contradictory results were encountered when evaluating the efficacy of hip bracing. In a prospective study comparing hip bracing with physiotherapy as opposed to physiotherapy only, Newcomb et al. [42] found no differences in patient-reported outcomes (NRS pain, iHot-33 and HAGOS questionnaires) after 4 weeks of follow-up. Patients reported discomfort when wearing a brace (4.8/10 on NRS (numeric ratio scale). Although participants did not cease to wear the brace, there were one or more adverse events for each participant, the most common of which were knee irritation of the braced leg (12/17, 71%), slipping of the brace (8/17, 47%), brace-related discomfort during or after brace wear (6/17, 35%), and contralateral hip pain (4/17,24%) [42]. As opposed to these findings, Eyles and colleagues [20] achieved significantly better PROMs when compared with a physiotherapy-only cohort at 6 weeks of follow-up. However, the intervention also involved concomitant oral consumption of NSAIDs and corticosteroid injections. Although the authors reported a similar rate of usage between groups, the impact of hip bracing as an individual intervention is questionable. Even if considered effective, further research is required to show whether it provides any benefit beyond short-term follow-up.

Another subject of debate is the efficacy of injections with hyaluronic acid. Abate et al. reported slight improvement in PROMs from pre- to post-intervention at 12 months. The concomitant activity modification and consumption of oral NSAIDs raises concerns about the validity of the conclusion of the study. Lee et al. [34] compared hyaluronic acid injections with corticosteroid injections and concluded after a mean follow-up of 3 months that no differences could be reported when injections were performed separately and individually. Ometti et al. [44] were the only to register significant improvement amongst all PROMs (visual analogue scale (VAS), Harris Hip Score (HHS), Lequesne) at 12 months after using a derivative of HAc. Recent research provides promising data regarding the potential of high molecular weight HAc injections as shown in a meta-analysis for hip osteoarthritis by Ebad Ali et al.[18] The authors concluded that short-term pain relief may be obtained in patients with hip degeneration.

A more valid modality for pain relief is injection with corticosteroids, as per a systematic review and meta-analysis by Zhao et al. [53] The authors compared all types of injection including HAc, corticosteroids, and platelet-rich plasma (PRP) for hip OA. When compared to our results, in the reports by Pennock [46] and Lee [34], corticosteroid injections achieved similar outcomes when compared to HAc injections, activity modification and hip arthroscopy. However, three studies [14, 32, 49] have mentioned a high proportion of patients undergoing surgery (from 50 to 100%) within 12 months after injection. These findings may support the hypothesis that the corticosteroid injection can provide short-term pain relief and possibly delay the need for THA, but do not ultimately alter the course of the disease process.

The limitations of the scoping review are directly linked to the limitations of the included studies. No evidence on outcomes for non-operative management for FAI with Tönnis Grade 2 or more OA was found. Studies that report on outcomes for these patients may have described the indication as hip OA, without describing the primary aetiology that may have been different from FAI (dysplasia, osteonecrosis, Perthes, posttraumatic or other) [23]. This did not allow inclusion of these studies for further assessment.

A lack of evidence of outcomes for non-operative management for patients suffering from FAI and concomitant Tönnis Grade 2 OA was found. Along with the findings of a recent systematic review [3] where inconclusive and contradictory evidence of outcomes of hip arthroscopy in this patient cohort were described, the scientific knowledge about the management of these patients remains insufficient and therefore lays the foundation for a randomised controlled trial. Although a THA may be a successful intervention in this specific cohort of patients with promising outcomes [31, 38], further research is warranted in determining the efficacy in delaying the need for a THA.

## Conclusion

There is a lack of evidence of outcomes following non-operative management of FAI with concomitant Tönnis Grade 2 or more OA of the hip. Further studies are needed and should explore the non-operative interventions that were employed for FAI and milder OA.

For mild OA, there is strong evidence for a hip-specific physiotherapy programme including activity modification and core strengthening exercises. Adjunct interventions such as corticosteroid injections and NSAID consumption may be valuable in delaying the need for surgery.

## Supplementary Information

Below is the link to the electronic supplementary material.

Supplementary file1 (DOCX 15 KB)

Supplementary file2 (DOCX 28 KB)

## Data Availability

The raw data are available upon request to the corresponding author.
