# Supplementary material for: No evidence exists on outcomes of non-operative management in patients with femoroacetabular impingement and concomitant Tönnis Grade 2 or more hip osteoarthritis: a scoping review
Source: Knee Surg Sports Traumatol Arthrosc. 2022 Dec 9;31(6):2103–22. doi: 10.1007/s00167-022-07274-y (PMC10183431; doi:10.1007/s00167-022-07274-y)
Supplement: Supplementary file 1 — Supplementary file1 (DOCX 15 KB) [file 167_2022_7274_MOESM1_ESM.docx]

**Supplementary Table 1.** Search terms used for scoping review

| **Combinations of Key-Words** |
| --- |
| (((conservative and (femoroacetabular impingement)) OR (conservative and (FAI)) OR (physiotherapy and (femoroacetabular impingement)) OR (physiotherapy and (FAI)))  (((nonoperative and (femoroacetabular impingement)) OR (nonoperative and (FAI)) OR (injection and (femoroacetabular impingement)) OR (injection and (FAI))) |
