# Supplementary material for: No evidence exists on outcomes of non-operative management in patients with femoroacetabular impingement and concomitant Tönnis Grade 2 or more hip osteoarthritis: a scoping review
Source: Knee Surg Sports Traumatol Arthrosc. 2022 Dec 9;31(6):2103–22. doi: 10.1007/s00167-022-07274-y (PMC10183431; doi:10.1007/s00167-022-07274-y)
Supplement: Supplementary file 2 — Supplementary file2 (DOCX 28 KB) [file 167_2022_7274_MOESM2_ESM.docx]

| **Supplementary Table 2**. Physiotherapy Intervention Breakdown | | | | | | | | | |
| --- | --- | --- | --- | --- | --- | --- | --- | --- | --- |
| ***Study*** | L**oE** | **Nr** | **Follow-up (months)** | **Intervention** | **Exercise Scheme** | **Frequency and Duration** | **Adherence to exercise** | **PROMs pre to postop** | **Conversion to Surgery / THA** |
| **Casartelli 2019** | IV | 28 | 4 | Activity Modification;  Hip-specific strengthening, **core stability**, postural balance exercises | Supervised sessions lasted 45 to 60 min and included a warm up, 4 hip-specific strengthening exercises, 2 functional lower limb strengthening exercises, 2 core stability exercises, and 2 postural balance exercises. Home-based sessions lasted about 15 min and included a warm up, 1 hip abductor or extensor strengthening exercise, 1 functional lower limb strengthening exercise and 1 core stability exercise | 12 weeks with 4 sessions per week | 2 face-to-face sessions per week conducted at the clinic under the supervision of a therapist and 2 sessions per week at home.  Patients completed a protocol sheet after each supervised and home-based session | GTO: 9 (29%) much better; 7 (22%) better; 6 (19%) somewhat better; 5 (16%) unchanged; 4 (14%) worse. Total 16 (52%) better; 11 (55%) responders, 9 (45%) non-responders | 8 (25%) had hip surgery |
| **Emara 2011** | IV | 37 | 24 | Physiotherapy  Adaptation to pain-free ROM modification of daily activities | All patients underwent 4 stages of conservative treatment:   1. Avoid physical activity and the use of anti-inflammatory drugs (diclofenac 50 mg, twice a day) for 2 to 4 weeks during the acute attack. 2. Physiotherapy to improve hip external rotation and abduction in extension and flexion, and to avoid the ‘W’ sitting position. 3. Assessment of the normal ROM after the acute pain subsided. The patients were instructed to adapt to their safe range of movement 4. Modification of activities of daily living predisposing to FAI (hip internal rotation associated with flexion and adduction) | Physiotherapy for 2 to 3 weeks in the form of stretching exercises (20 to 30 minutes daily) | No strategies apart from clinical follow-up | mHHS improved (p<0.01); NAHS improved (p<0.01); VAS improved (p<0.01) | 4 (11%) resulted in surgical intervention |
| **Aoyama 2019** | III | 10 | 2 | Pelvic Floor Muscle; **Trunk Training**; Activity Modification | Pelvic Floor Muscle Training (hip abduction, buttock elevation, pelvic tilt exercises) + **Trunk Training (Plank and Bird Dog exercises**) + Activity Modification, | 20 minutes daily for 8 weeks | Compliance status was confirmed verbally and with documented records during every visit | Significant better improvement in study group with trunk exercises for Vail hip score and iHOT12 (p<0.001) | n.R. |
| *Control Group* | | 10 | 2 | Pelvic Floor Muscle; Activity Modification | Pelvic Floor Muscle Training (hip abduction, buttock elevation, pelvic tilt exercises) + Activity Modification | 20 minutes daily for 8 weeks |  |  | n.R. |
| **Pennock 2018** | II | 65 | 25.7 ± 6.7 (12.2-45.5) | Activity Modification | Therapeutic exercises for posture, training the patient to achieve neutral spine. Lumbo-pelvic mobility training. Core stabilisation techniques + Proximal strengthening + Lower extremity flexibility. | Daily for 6 weeks | Verbal confirmation of compliance at each clinical follow-up. | No significant differences were noted in the proportion of patients meeting the MCID in the mHHS among the 3 treatment groups. | n.R. |
| *Controls (1)* | | 11 | 25.4 ± 8.8 (11.7-40) | Corticosteroid Injection | Formal PT initiated 1 week after intervention. Running progression program 8-12 week. | Daily for 6 weeks | Verbal confirmation of compliance at each clinical follow-up. |  |  |
| *Controls (2)* | | 17 | 31.8 ± 12 (12.3-49.7) | Hip Arthroscopy | Formal PT initiated 1 week after surgery. Running progression program 8-12 weeks post-op. Full clearance to sport 4-6 months later. | Daily for 6 weeks | Verbal confirmation of compliance at each clinical follow-up. |  |  |
| **Guenther 2017** | IV | 20 | 3 | Physiotherapy. **Core exercises** | Phase 1: Optimal, pain-free activation of key muscle groups, improve neuromuscular strength + develop muscular endurance.  Phase 2: Build on patients’ foundation of muscle strength + increase exercise intensity.  Phase 3: Further increase hip muscle strength. | 4 times per week for 10 weeks (3 sets of 10-12 repetitions per exercise per session) | Consultation with the study kinesiologist during weeks 1,2,4,6,8 to ensure exercises were performed properly | HOOS: Pain: +8.5 (p=0.003); Symptoms: +7.9 (p=0.022); ADLs: +10.4 (p=0.003); Sports: +11.7 (p=0.003); QOL: +7.6 (p=0.025) | foreplaned surgery in all patients, 5/19 (26%) cancelled upcoming surgery after program |
| **Griffin 2018** | I | 195 | 12 | Individualized Physiotherapy;  Activity Modification | 4 components:  (1) Assessment of pain, function, and range of hip motion  (2) Patient education  (3) exercise programme that has features of individualisation, progression, and supervision  (4) help with pain relief, such as one X-ray or ultrasound-guided intra-articular steroid injection when pain prevents performance exercise. | 12-24 weeks with a physiotherapist | Patient completed exercise diaries + case report forms recorded by physiotherapists | iHOT33 improvement greater in HA group (p=0.009), no difference in other PROMs. More adverse events related to intervention in HA group (p=0.017). Physiotherapy more cost-effective at 12 months in the setting of UK costs  none | 14 (8%) received hip arthroscopy |
| *Control Group* | | 182 | 12 | Hip Arthroscopy | Patients referred to outpatient physiotherapists as per usual care for surgery. | Not specified | Clinical follow-up |  | 1 (1%) converted to THA after hip joint infection |
| **Hunter 2021** | I | 50 | 12 | Physiotherapy | 3 components:  (1) Personalised exercise programme supervised by physiotherapist.  (2) Education about the condition and management  (3) Advice regarding pain relief | 6-10 sessions with a physiotherapist over 6 months. | Logbook given to patients to record exercises completed at home. | Hip-related quality of life (iHOT-33) showed a statistically and clinically important improvement in arthroscopy of 14 units (p = 0.003)  n.R. | n.R. |
|  | | 49 | 12 | Hip Arthroscopy | Usual post-operative rehab protocol recommended by their surgeon. | Not specified | Clinical follow-up |  | n.R. |
| **Kekatpure 2017** | III | 54 | 27.5 | Activity Modification | Activity modification included avoiding squatting, leg crossing, pivoting, excessive physical activity, and sitting on the floor. | Twice a day for 6 weeks, and thereafter as required. | Clinical follow-up at 2, 6, 12 weeks. | The nonsurgical group had significant improvements  in all clinical scores at the end of follow-up (p < 0.001) | 44 hips underwent hip arthroscopy (45.4%) at 10 months (3 – 29.5) |
| *Control Group* | | 44 | 25.4 | Hip Arthroscopy | Patients underwent the same 3 months conservative treatment program prior to surgery. |  |  |  |  |
| **Kemp 2016** | III | 17 | n.R. | Personalized FAI-specific Physiotherapy | Hip joint manual therapy techniques, specific strength­ening exercises for the hip and trunk muscles + functional activity specific retraining. | 8 physical therapy treatment sessions over a 12-week period, and 12 weekly supervised gym visits. | Used the mobile phone exercise application Physitrack (Physitrack Ltd, London, UK) or paper-based training diaries. | All scores experienced improvement with more magnitude in the specialized group (iHOT-33: 27 ± 26; HOOS-QoL: 22 ± 18; HOOS pain: 20 ± 16)  n.R. | n.R. |
| *Control Group* | | 7 | n.R. | Standard Stretching Therapy | Hip joint manual therapy, muscle stretching, health education. |  |  |  | n.R. |
| **Mansell 2018** | I | 40 | 24 | Physiotherapy | Motor control exercises (reverse lunge, Romanian deadlifts, lateral step-down, side plank, hip flexion) + Manual therapy approach (internal rotation, FABER self-mobilisation with band, prone figure-4, self-myofascial release) | Two 45min physical therapy visits per week for 6 weeks (12 sessions total). | Self-efficacy was assessed with the Self-Motivation Inventory (40-itme tool). | Statistically significant improvements were seen in both groups on the HOS and iHOT-33, but the mean difference was not  significant between the groups at 2 years  n.R. | 28 (70%) underwent surgery at a mean of 6.5 months |
| *Control Group* | | 40 | 24 | Hip Arthroscopy | Evaluated by a physiotherapist within 7 days post-op to begin rehab. FABER, internal rotation, quadruped rock. | Post-operative rehab completed within 6 months following surgery. |  |  | hip fracture (n=1); heterotopic ossification (n=1); revision surgery (n=5); THA (n=1). |
| **Martin 2021** | I | 44 | 12 | Physiotherapy | Core-based physiotherapy focused on normalising gait, optimising range of motion, and strength training. | 24 weeks | Clinical follow-up to pass a predefined ‘criteria for advancement’ | Intention-to-treat analysis revealed significantly greater iHOT-33 scores (112.11; P = .007) and mHHS scores (16.99  points; P = .04) in the surgical group than the physiotherapy group at 12 months  postoperative physical therapy | 28 (63.6%) patients went on with hip arthroscopy at a mean of 6 months (4 – 8) |
| *Control Group* | | 44 | 12 | Hip Arthroscopy | Standardised postoperative physical therapy protocol including gait training, core control, stair training, and training in translational movements | 24 weeks | Clinical follow-up to pass a predefined ‘criteria for advancement’ |  | n.R. |
| **Narveson 2018** | IV | 6 | 2.5 (2 – 3) | Physiotherapy | Restore normal kinaesthetic awareness to the joint (single leg balance, dynamic balance) + functional movement (hip hinge, squat, posterior lunge) | 68-91 days (mean 81 days) | Clinical follow-up using flexibility testing and functional assessment | Clinically important improvements were seen on all self-reported outcome measures (iHOT – 33, NRS and other) | 0 at 24 months |
| **Palmer 2019** | I | 110 | 8 | Physiotherapy Activity modification | Muscle strengthening to improve core stability and movement control. Avoid impingement positions (extremes of hip flexion, abduction, internal rotation). | 8 sessions over 5 months | Compliance recorded by the treating physiotherapist at each follow-up. | After adjusting for baseline HOS ADL, age, sex, and study site, the mean HOS ADL was 10.0 points higher (6.4 to 13.6) in the arthroscopic hip surgery group compared with the physiotherapy programme group (p<0.001))  postoperative physical therapy | 2 (2%) at 8 months |
| *Control Group* | | 112 | 8 | Hip Arthroscopy | Postoperative physiotherapy, provided as routine care in the NHS. This focused on maintaining range of movement and a graduated return to activity. | Not specified |  |  | none |
| **Spencer 2017** | III | 36 | 20 (12–30) | Physiotherapy, Corticosteroid Injection or both | Patient education, avoidance or proactive manoeuvres, and use of anti-inflammatory medications. | 3 months | Clinical follow-up | Mean mHHS scores similarly showed little change in the non-operative group (P¼ 0.91), and improved in the HA group (p<0.001). At final follow up, mean NAHS scores after HA were significantly higher than scores for waitlist patients (p<0.001).  n.R. | n.R. |
| *Control Group* | | 36 | 18 (12–36) | Hip Arthroscopy | 3 months conservative treatment with physiotherapy before surgery. | 3 months | Clinical follow-up |  | n.R. |
| **Wright 2016** | I | 7 | 2 | Manual therapy and supervised exercise; Advice and home exercise | Muscle strengthening, muscle stretch, and neuromuscular/motor control exercise to normalise hip alignment. | Twice per week for 6 weeks. | Following a ‘hip handout’ sheet and clinical follow-up | The between group differences for changes in pain or physical were not significant. Both groups showed statistically significant improvements in pain  n.R. | Eight out of 15 (53%) patients elected to proceed with surgery |
| *Control Group* | | 8 | 2 | Advice and home exercise alone | Avoidance of long-term sitting, hip strengthening exercises. | *Ad libitum* | Following a ‘hip handout’ sheet. |  |  |
| **Zogby 2021** | II | 50 | 61.5 ± 8.2 (43.4-74.9) | Physiotherapy, Activity Modification | Avoid hip flexion, core stabilisation (rotational stability exercises, transverse abdominus recruitment, multifidus recruitment) + proximal strengthening (single leg balance + lunges) + lower extremity flexibility. | 6 weeks | Clinical follow-up | Hips treated with activity modification and physical therapy alone met the MCID for the mHHS at a rate of 74% compared with a 71% rate for hips treated with an injection, and a 75% rate for hips treated with arthroscopic surgery. No difference in the proportion of hips that met the MCID for the mHHS based on treatment course was observed (p = .99).  Activity modification involved discontinuation of all sports and activities that involved running, jumping, or high hip flexion | 12 (24%) underwent hip arthroscopy at a mean of 9.2 months (range, 1.3-18.1) |
| *Control Group* | | 12 | 62.3 ± 7 (45.5-76.4) | Hip Arthroscopy | Physical therapy initiated 1 week after surgery. Running progression program around 8-12 weeks after surgery. | 12 weeks | Clinical follow-up |  | n.R. |
| Nr – number of hips; Avg – average; n.R. – not reported; n/a – not available or extracted not possible; LoE – level of evidence; OA – osteoarthritis; BMI – body mass index; FAI – femoroacetabular impingement; M – male; F – female; PROMs – patient-reported outcomes; THA – total hip arthroplasty; GTO – global treatment outcome; HOOS - Hip disability Osteoarthritis Outcome Score; mHHS – modified Harris Hip Score; VAS – visual analogue scale; iHOT – International Hip Outcome Tool; NRS – numeric pain rating scale; ROM – range of motion; NSAIDs – non-steroidal anti-inflammatory drugs; MCID – minimal clinically important difference; QoL – quality of life; ADL – activity of daily living | | | | | | | | | |
